# Reactive laser interference patterning on titanium and zinc in high pressure CO_2_

**DOI:** 10.1038/s41598-022-19916-9

**Published:** 2022-09-21

**Authors:** Amandeep Singh, Tero Kumpulainen, Kimmo Lahtonen, Saara Söyrinki, Jorma Vihinen, Erkki Levänen

**Affiliations:** 1 Materials Science and Environmental Engineering, Tampere University, Tampere, Finland; 2 Automation Technology and Mechanical Engineering, Tampere University, Tampere, Finland; 3 Faculty of Engineering and Natural Sciences, Tampere University, Tampere, Finland; 4 VTT Technical Research Centre of Finland Ltd, Helsinki, Finland

**Keywords:** Materials science, Nanoscience and technology, Optics and photonics, Physics

## Abstract

Direct laser interference patterning (DLIP) is a versatile technique for surface patterning that enables formation of micro-nano sized periodic structures on top of the target material. In this study, DLIP in high pressure, supercritical and liquid CO_2_ by 4-beam DLIP was used to pattern titanium and zinc targets. Field emission scanning electron microscopy, atomic force microscopy, and X-ray photoelectron spectroscopy was used to characterize the patterned surfaces. Field emission SEM analysis showed presence of ordered uniform donut ring pattern with hollow centers for both titanium and zinc with a period slightly under 3 µm while topographical images from atomic force microscopy revealed donut rings protruding outwards typically around 200 nm from target surface and consisted of a crevice at the center with a depth typically around 300 nm and 250 nm for titanium and zinc target, respectively. Based on X-ray photoelectron spectroscopic analysis, this is the first study to report formation of TiO_2_, TiC, ZnCO_3_, and zinc hydroxy carbonate on the pattern by DLIP in supercritical and liquid CO_2_ for titanium and zinc targets. Pressurized CO_2_ is demonstrated as a promising environment with mirror-based DLIP system for reactive patterning. Due to the superior transport properties and solvent power of supercritical CO_2_, the current study opens possibilities for reactive patterning in environments that may not have been previously possible.

## Introduction

Surface engineering of materials to create micro and nanostructures for surface enhanced properties has gained interest due to its association with important contemporary topics such as climate change, sustainability, safety, health care, and materials performance. Laser treatment of surfaces has been demonstrated to form antireflective, superhydrophobic, and anti-bacterial surfaces, and further to make biomedical implants, 3D scaffolds for cell cultures and tissue engineering, and in photonics to form ordered long-range quantum dots^1–7^.

While present direct laser writing/patterning of material surfaces has been successfully demonstrated to produce morphologies with feature sizes between 10 and 15 µm, this technique has limitations in terms of pattern size (below 5 µm) and patterning process speed^8^. If the goal is finer ordered pattern and faster patterning process, one solution is direct laser interference patterning/lithography (DLIP) technique in which two or more laser beams interfere at target surface to cause formation of subwavelength sized structures/patterns. There can be different optical setups in DLIP to accomplish desired pattern size, shape, and morphology. DLIP has been demonstrated as a promising surface engineering tool for producing long-range well-defined high-resolution surface patterns with high throughput^9^. 2–5 beam DLIP is commonly used to grow patterned materials, such as in molecular-beam epitaxy reactors for nanolithography^10–12^. DLIP has mostly been demonstrated in vacuum or in low-pressure reactive gases environment. The reactive gas environment plays a significant role in DLIP. CO_2_ is an inert gas at room temperature and can also be used as a carrier of reactive species during laser irradiation process at high pressures and high local temperatures^13^. As a supercritical fluid, CO_2_ is known to have superior transport properties for species dissolved in it and also for its high solvent power, which therefore allows addition and dissolution of co-solvents that may not otherwise be soluble, to create novel processing environments during DLIP. Furthermore, the solvent power can easily be controlled simply by changing its pressure or temperature. However, CO_2_ exists in supercritical state over 73.8 bar pressure and 31.1 °C temperature^14^. Interference system for high-pressure, high-density media has not been previously possible. High-pressure, high-density media poses practical challenges for interference, such as distortion of laser beam, optical fluctuations, vibrations, and reflection from optical viewports. Recently, DLIP in high pressure CO_2_ was first demonstrated^15^.

In that study, laser interference in liquid, gas, and supercritical CO_2_ to form patterned microstructures on zinc sputtered on glass, titanium, and thin film coating on aluminium was demonstrated. For this, two different optical approaches were used; a lens based, and a mirror-based DLIP. The pattern consisted of ordered structures with size dependent on the type of optical system used. Whether the patterns consist of only native metal from target or are other reaction species involved, remains largely unknown. However, previous studies in pulsed laser ablation (PLA) may indicate what can be expected. Singh et al., reported previously on the synthesis of titanium dioxide nanoparticles from titanium by PLA in scCO_2_ where the key result was oxidation of titanium from the oxygen formed by decomposition of scCO_2_^13^. In other words, CO_2_ was utilized for production of titanium dioxide nanoparticles from titanium. In another study, the synthesis of TiO_2_ nanoparticles with carbon shell was demonstrated by laser ablation of titanium bulk target in scCO_2_ where the oxidation of titanium to TiO_2_ nanoparticles and presence of carbon film was reportedly due to CO_2_ decomposition^16^. While presence of oxygen and carbon are reported for nanoparticles, there are no studies on oxidation of laser irradiated target surface by scCO_2_, to the best of our knowledge. Can similar phenomenon be observed in thin film targets compared to observations in nanoparticles? The current study aims to answer this research question as well.

In this study, we demonstrate DLIP on titanium and zinc targets in high pressure environment in scCO_2_ and liquid CO_2_ at 100 bar pressure to synthesize patterned surfaces. Data in the study indicated CO_2_ as a reactive medium that participates in the reactions with the target metal and leads to the formation of compounds such as oxides, carbide, and carbonate on the patterned area.

## Results

In this section, we present field emission scanning electron microscopy (FESEM) images, atomic force microscopy (AFM) 2D and 3D topographical images that show protruding donut ring pattern formed by DLIP on titanium and zinc in pressurized CO_2_. Analysis of such pattern and elemental composition in pressurized medium by DLIP has not been reported earlier. Furthermore, X-ray photoelectron spectroscopy (XPS) and X-Ray induced auger electron spectroscopy (XAES) results are presented that indicate formation of Ti-based and Zn-based oxide, carbide, carbonate, and hydroxy carbonate compounds on the pattern.

### Single pulse DLIP on titanium in supercritical and liquid CO_2_

**Figure 1:**
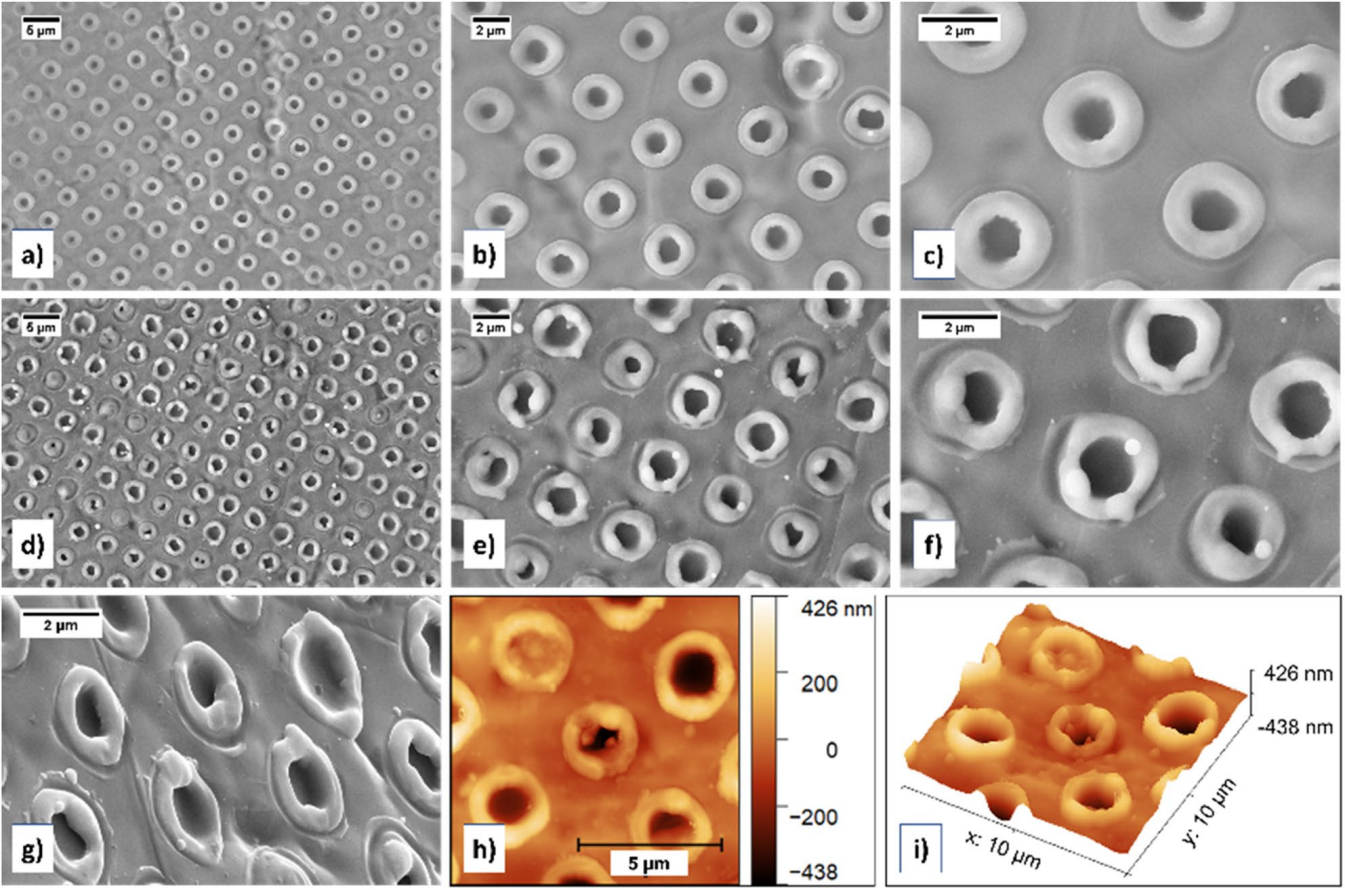
FESEM micrographs for DLIP on titanium in scCO_2_ (**a**–**c**) and liquid CO_2_ (**d**–**f**) at 100 bar pressure. Presence of ordered donut/ring pattern was observed for both environments, with only slight difference. Whereas in scCO_2_, the donut pattern seemed smooth, in case of liquid CO_2_, the donut pattern consisted of melt deposits on top of the donut rings that were sometimes spherical. (**g**) Tilt-view FESEM micrograph revealed donut rings formed by DLIP in scCO_2_ protrude out of the target plane and contain a crevice in the centre. Atomic force microscopy (AFM) image showing topographical (**g**) 2D and (**h**) 3D images of donut ring pattern on titanium.

Single pulse DLIP was performed on titanium target in scCO_2_ at 100 bar pressure and 35 °C temperature. Microscopy analysis of titanium target with FESEM revealed presence of ordered dot pattern (Fig. 1a). The dot pattern shape at higher magnifications was observed to be in the form of donut like rings (Fig. 1b,c), with a hollow centre. Similar, yet coarser pattern structures have previously been demonstrated, however not in pressurized medium^17^. Higher magnification micrograph (Fig. 1c) revealed presence of some deposited nanoparticles on the laser patterned area, indicating presence of ablation like phenomenon that formed nanoparticles. During the single pulse DLIP experiment, a visible plasma flash was observed, which indicated that the laser fluence at the pattern was higher than the ablation threshold of the titanium target.

Then another single pulse DLIP experiment was performed on titanium target, however in liquid CO_2_, at 100 bar pressure and 27.5 °C temperature. FESEM micrographs (Fig. 1d,e) revealed ordered dot pattern with period slightly under 3 µm, consisting of slightly asymmetrical donut rings with hollow centres. At higher magnification, the thermal effects of the nanosecond laser used were evident in the FESEM micrograph (Fig. 1f) due to the presence of surface features on top of the donut pattern corresponding to melting, solidification phenomenon. This is explained further in the discussion section. FESEM-EDS analysis of DLIP patterned titanium targets in scCO_2_ indicated that carbon and oxygen could be present. The donut ring thickness measured from FESEM images ranged from 661 to 903 nm and typically around 750 nm.

Tilt-view FESEM imaging further improved the understanding of the shape of the pattern. The tilt-view FESEM micrograph of the pattern (Fig. 1g) showed a crevice in the centre part of the pattern while the edges were raised outwards to form a ring pattern. This topography was further elucidated using AFM topographical 2D and 3D images of pattern on titanium (Fig. 1h,i). Based on the AFM measurements, the donut ring height in case of titanium was typically around 200 nm varying between 130 and 320 nm depending on the region on the laser patterned surface while the pit depth at the center of the donut rings was typically around 300 nm.

### Single pulse DLIP on zinc in supercritical and liquid CO_2_

**Figure 2:**
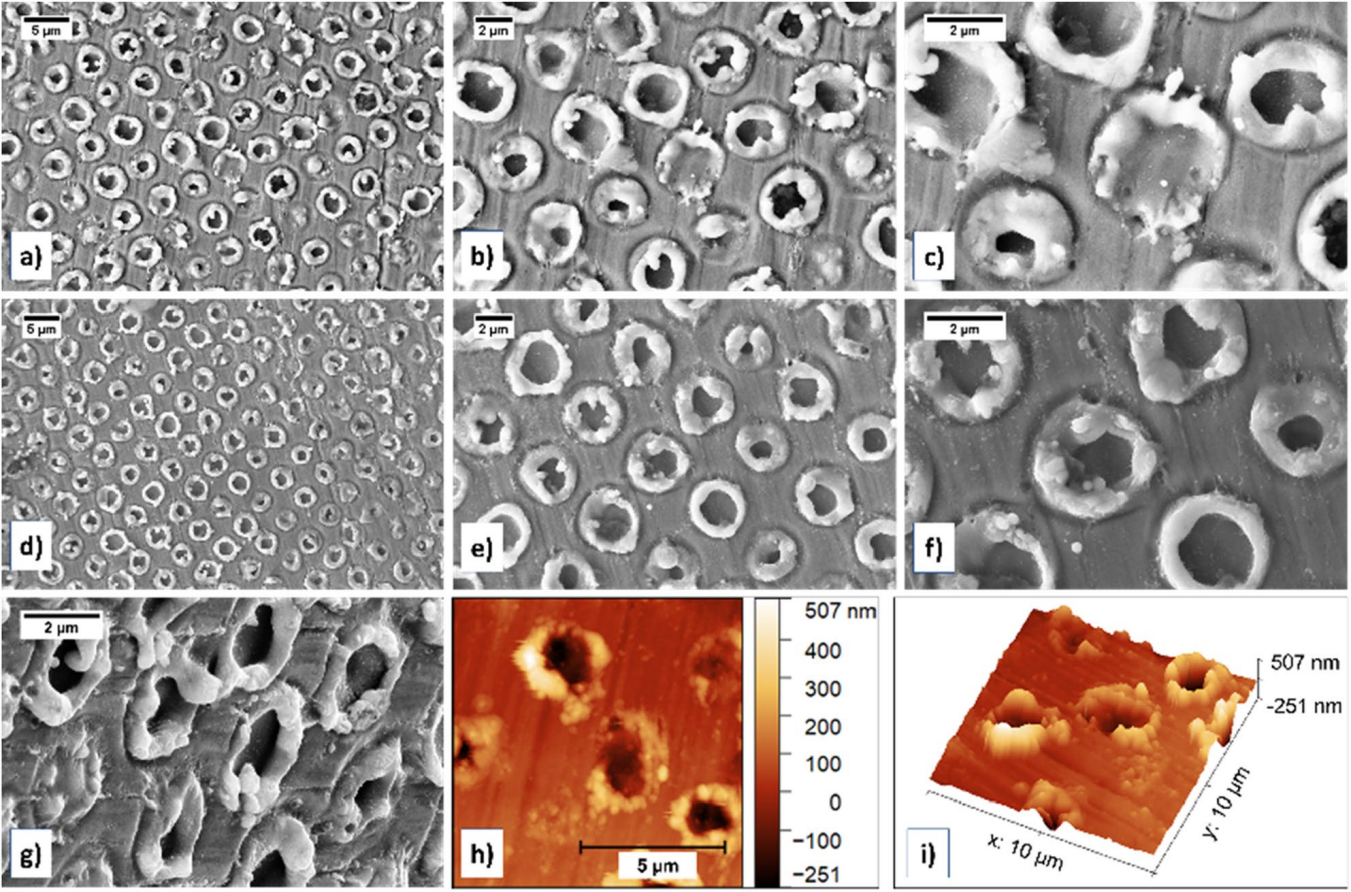
FESEM micrographs for DLIP on zinc in scCO_2_ (**a**–**c**) and liquid CO_2_ (**d**–**f**) at 100 bar pressure. Presence of round ordered structures donut/ring pattern was observed for both environments, without any significant difference. (**g**) Tilt-view FESEM micrograph revealed protruding slightly irregular surface on donut rings on the zinc target formed by DLIP in scCO_2_ with a crevice in the centre. Atomic force microscopy (AFM) image showing topographical (**g**) 2D and (**h**) 3D images of donut ring pattern on zinc.

Single pulse DLIP was performed on zinc target in supercritical CO_2_ at 100 bar pressure and 35 °C temperature, and in liquid CO_2_ at 100 bar and 27.5 °C. FESEM micrographs for DLIP patterned Zn in scCO_2_ (Fig. 2a–c) and Zn in liquid CO_2_ (Fig. 2d–f) indicated presence of ordered donut-like ring structures, however not as regular shaped rings as observed for titanium (Fig. 1a–c). Unlike in case of titanium sample, for zinc sample the patterns rings were not as smooth and ordered for the same process parameters. This is understandable since for laser-matter interaction with a nanosecond laser, the thermal properties of the material is a more crucial factor as nanosecond laser interaction involves mostly photothermal melting and evaporation mechanisms^18–20^. The difference in material properties then result in different pattern. The laser-induced melting caused material flow on pattern. In case of zinc in scCO_2_ and liquid CO_2_, micrographs showed ring pattern with irregular pointy edges with melt spheres. Further, the nanoparticles ablated and redeposited on the pattern surface were observed and in somewhat more amount than in case of titanium. The pattern formation likely involved melting, solidification, and deposition, based on pattern appearance (Fig. 2b,c,e,f). Similar to the titanium sample, in zinc too, FESEM-EDS analysis indicate possibility of presence of carbon and oxygen. The donut ring thickness measured from FESEM images ranged from 395 to 1300 nm and typically around 800 nm.

While the ring pattern consisted of smooth round edges in titanium samples, rough slightly irregular rings in zinc samples were observed in the tilt-view FESEM micrograph (Fig. 2g). In some cases (such as in Fig. 2c,d,g,h), the dot pattern did not form the regular donut ring with a central hole type of pattern for every donut-ring formed. AFM 2D and 3D topographical images from such an area revealed similar protruding rings with a crevice in the centre. This is a result of the modulation of pattern intensity due to the presence of fringe over the four-beam intensity pattern^15^. The AFM data for zinc sample revealed the donut ring height was typically around 180 nm with variation between 67 and 440 nm while the pit depth was typically around 250 nm.

### X-ray photoelectron spectroscopy of patterned zinc and titanium

XPS further corroborated EDS observation on the presence of carbon and oxygen. Table 1 shows the relative elemental surface composition (atomic %) of laser patterned area in zinc and titanium samples. The main elements were C, O, Zn (in zinc sample), and Ti (in titanium sample), as expected. Also, higher amount of N was detected in the titanium sample compared to the zinc sample. In addition, traces of other elements were detected: Si and Ca in both zinc and titanium samples, S and Cl in the zinc sample, as well as K and Zn in the titanium sample.

**Table 1:** Relative elemental surface composition (at.%) of laser patterned areas in Zn and Ti samples based on XPS.

Element	Zn laser patterned	Ti laser patterned
C	44.61	37.44
O	40.40	33.87
Ti	–	20.25
Zn	10.42	0.06
N	0.69	5.63
Si	0.89	0.76
S	0.85	–
Cl	1.23	–
K	–	0.58
Ca	0.91	1.41

**Figure 3:**
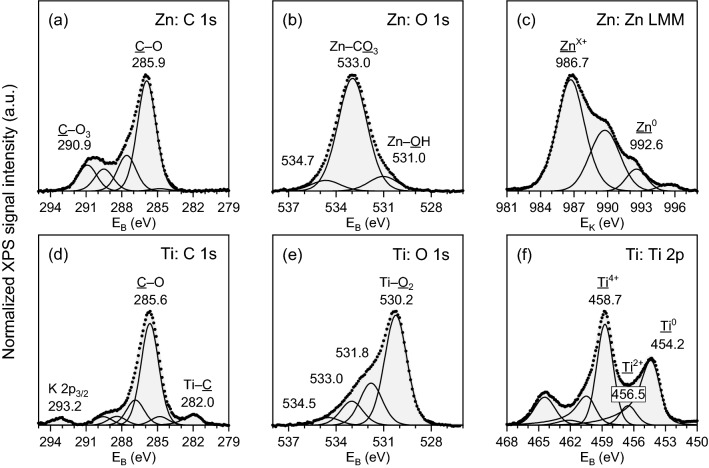
The XPS/XAES core level spectra of Zn sample: (**a**) C 1s, (**b**) O 1s, (**c**) Zn LMM, and Ti sample: (**d**) C 1s, (**e**) O 1s, (f) Ti 2p.

The main C 1s component on both samples (Fig. 3a,d) was low oxidation state C–O species at ~ 286 eV accompanied by some higher oxidation state C–O_x_ species between 287 and 289 eV. In addition, the zinc sample contained high binding energy C 1s component at 290.9 eV, most likely corresponding to CO_3_ which may be bonded to Zn^21^. Some Ti carbide at 282.0 eV was detected only on the laser patterned area of the titanium sample.

The lowest binding energy O 1s component on the zinc sample (Fig. 3b) was detected at 531.0 eV which is too high value for pure ZnO, further indicating that Zn is mostly bonded to OH and/or CO_x_. The main O 1s peak detected at 533.0 eV corresponds e.g. to Zn–CO_3_^21^. The concentration of the O 1s at 533.0 eV (33.01 at.%) is about three times that of Zn 2p_3/2_ at 1023.2 eV (10.42 at.%) matching a stoichiometry of Zn_1_CO_3_. Further, the O 1s peak at 531.0 eV indicates possible presence of Zn–OH. The concentration of O 1s at 531.0 eV (4.28 at.%) is about half of that of Zn. These ratios would match stoichiometry of Zn_2_(OH)_1_(CO_3_)_2_. However, at the same time large part of O 1s signal originates also from other C–O species not bonded to Zn. If assuming C–O–C species in C 1s at 285.9 eV, this would indicate stoichiometry of Zn species more towards Zn_2_(OH)_1_(CO_3_)_1_.

The laser patterned zinc sample contained Zn at two different chemical states in the ratio Zn^0^/Zn^X+^ = 1:9. The metallic substrate Zn LMM signal (Fig. 3c) was detected at E_K_ = 992.6 eV and oxidized Zn LMM signal at E_K_ = 986.7 eV which is lower kinetic energy than typically reported for pure ZnO, again matching Zn bonding to OH and/or CO_x_. Besides Zn_x_(OH)_y_(CO_3_)_z_ the surface may contain traces of Zn_2_SiO_4_, ZnSO_4_, ZnCl_2_, and other stoichiometries of ZnCO_x_.

The laser patterned titanium sample contained Ti at three different chemical states: Ti^0^ at 454.2 eV, Ti^2+^ at 456.5 eV, and Ti^4+^ at 458.7 eV, in the ratio Ti^0^/Ti^2+^/Ti^4+^ = 5:1:5. The main core level components of Ti 2p_3/2_ (458.7 eV, 9.28 at.%) (Fig. 3f) and O 1s (530.2 eV, 20.29 at.%) (Fig. 3e) match pure TiO_2_ oxide in energy and intensity ratio. In addition, there was a smaller Ti^2+^ component in Ti 2p which may likely originate from mixed titanium oxide and nitride phases Ti_x_O_y_N_z_ corresponding to the main N 1s peak detected at 396.7 eV.

As a summary, O and C are present in Titanium as TiO_2_, TiC, and TiO_x_N_y_, while in Zinc they are likely present mostly as ZnCO_3_ and/or Zn_x_(OH)_y_(CO_3_)_z_. XPS data suggests scCO_2_ participates in laser interaction with the target materials during DLIP for both Ti and Zn.

## Discussion

Before the discussion on the morphology of pattern and the role of pressurized CO_2_, the observation of some inhomogeneities in some parts of the pattern is discussed. Even in low pressure or ambient pressure atmosphere, 4-beam DLIP is challenging and prone to misalignment of optics which introduce periodic modulations of the intensity profile of the interfering beams^22^. However, utilizing a top-hat laser beam intensity distribution may improve uniformity of the pattern to some extent, when compared to a conventional Gaussian laser beam intensity distribution^22^. In case of pressurized fluids too, slight modulation of pattern intensity is possible which may cause some inhomogeneity in the synthesized pattern.

### Discussion on the morphology of pattern

During DLIP process, the target material interacts with the laser beam at the local maxima of interference pattern. In case of nanosecond laser, the thermal mechanisms dominate and heating of the local maxima locations by Marangoni convection and recoil vapour pressure results in metallurgical processes such as melting, evaporation, ablation, and crystallisation^23,24^. This gives rise to an ordered structure at the local maxima positions. The donut-ring with hollow centres correspond to these local maxima positions. The hollow centres correspond to the regions with the highest local fluence, which therefore causes easy material removal. Further, the removed material forms the rim of the donut-ring pattern on the lower local fluence region^25^. The pattern results from two processes: (a) melting, and (b) melt expulsion from the centre of local maxima positions. First melting occurs at the target, and as the melt depth increases, the melt expulsion process starts and causes radial outflow of melt from centre of laser spots to form a rim of resolidified material around the spot edge^23^. This is the dominant mechanism of pattern formation observed in the FESEM and AFM images in the Figs. 1 and 2. The topography observed in the AFM 2D and 3D topography images in Figs. 1h,i, 2h,i of donut ring pattern protruding out from the target plane follow well to the two aforementioned pattern synthesis processes. Further, in Fig. 1c,e,f, an additional external shallow rim around the donut ring pattern was observed. This could either be the outer diameter of the local maxima spots or due to modulation of laser beam energy that caused surface instabilities. In the first case, the use of top-hat beam could mean that around the diameter of local maxima spots, the fluence may only be slightly lower than at centre so as not to cause any melt-expulsion but the laser damage to target material is visible. In the latter case, surface instabilities can happen due to interference of incident light with the reflected/refracted light from target surface, causing non-uniform energy input and modulated distribution of energy on the target surface^26–28^.

### Discussion on the role of DLIP medium—pressurized CO_2_

The state of the ambient medium is important in the patterning process. The plasma formed upon DLIP on the target is different at low pressures such as in gases compared to liquids. Liquid confinement holds plasma in a high-density state and results in condensation of ablated material^29^. Plasma formation upon interaction of nanosecond laser with target material inside liquids is well understood^30^. While there are some plasma studies for scCO_2_^31,32^, the plasma dynamics are not as well understood in the supercritical regime compared to in liquids. However, studies report a very rapid formation of plasma and CO_2_ decomposition over a timescale of few hundred nanoseconds upon laser irradiation, with plasma temperature between 3873 and 4873 °C^31,32^. CO_2_ breaks down into atomic oxygen^31,33^, and carbon ions and radicals^31^, as a result of high plasma temperature. Hot atoms in the titanium and zinc melt can then interact with reactive species formed upon CO_2_ decomposition. This results in presence of oxygen and carbon at the patterned surface, as observed in the XPS spectra for Ti and Zn. Such observations were previously only reported for nanoparticles synthesized by pulsed laser ablation in pressurized CO_2_ environment, not for target materials^16^. Laser interaction with titanium in high pressure CO_2_ forms mostly TiO_2_, while carbon may deposit on top^13,16^. However, if local temperatures at the laser spot exceed 2273 °C, in an environment with carbon, TiO_2_ can reduce to form TiC, although the conversion usually involves formation of lower oxidation state titanium oxides such as Ti_3_O_5_ and Ti_2_O_3_^34^. In the XPS spectra, peaks corresponding to both TiC (Fig. 3d) as well as lower oxidation state Ti such as Ti^2+^ at 456.5 eV (Fig. 3f) are observed. For Zn, ZnCO_3_ forms as it interacts with scCO_2_, although in the presence of H_2_O, zinc hydroxide carbonate can be formed^35,36^. This explains the higher carbon amount observed in the XPS relative elemental surface composition in Table 1 for zinc sample compared to titanium.

Based on above discussion, pressurized CO_2_ is demonstrated as a promising medium during reactive DLIP. Further, another key result is that the demonstration of DLIP in scCO_2_ for titanium and zinc opens several possibilities to add co-solvents (due to high solvent power of scCO_2_) for different pattern composition that may not have been previously possible.

## Conclusion

In the current study, we demonstrated mirror-based 4-beam single pulse DLIP on thin film titanium and zinc in scCO_2_ and liquid CO_2_ at 100 bar pressure. We report formation of ordered uniform donut ring pattern with a period slightly under 3 µm consisting of hollow centres for both titanium and zinc in both media, scCO_2_ and liquid CO_2_. The pattern formation process involves both Marangoni convection and recoil vapor pressure to cause heating of the local maxima positions. This is followed by melt and melt expulsion phenomenon, which causes the center of the local maxima position to expel the target material radially outward, which then forms the rim, also called donut-ring pattern in this study. This explained the shape of the observed pattern, which also corroborated the observation in the FESEM micrographs, and in the AFM 2D and 3D topography images wherein the donut rings appear to be protruding out of the target plane around 200 nm and mostly consist of a hollow center with the depth around 300 nm for titanium and 250 nm for zinc. The modulation of pattern intensity by interaction of incident light with light reflected/refracted from target surface caused some pattern positions not to have the ideal donut ring pattern. Further, change in the state of CO_2_ from scCO_2_ to liquid, and the difference in thermal properties of target materials resulted in slightly warped donut-ring pattern in some cases. Such variations resulted in wider range of ring thickness and ring height in the zinc target compared to the titanium target. Based on X-ray photoelectron spectroscopic analysis, the patterned area consisted of mostly TiO_2_, TiC, and TiO_x_N_y_ in case of titanium while ZnCO_3_, and zinc hydroxy carbonate for zinc.

As a conclusion, pressurized CO_2_ is demonstrated as a promising medium for reactive patterning with our DLIP system. The superior solvent power and transport properties of supercritical CO_2_ with co-solvents and easily tunable properties by changing temperature and pressure, enables new environments for DLIP to get the desired pattern composition. Future work will focus on the effect of co-solvents as well as on determining the difference between the pattern composition in air and scCO_2_ as DLIP environments and whether patterning in scCO_2_ could lead to any localized oxidization or carbonization. Nanostructured functionalized surfaces can improve the performance of materials for clinical applications such as for modulating cellular function via surface topography, as well as for imaging, diagnostics, and tissue engineering^37,38^. The patterned area on the target is small at around 4.5 mm in diameter, making the scalability challenging. However, scalability solutions are possible in similar ways as innovated in the field of pulsed laser ablation in liquids and/or employing a fixed optical design and laser but with the target mounted on a rotating target holder.

## Methods

**Figure 4:**
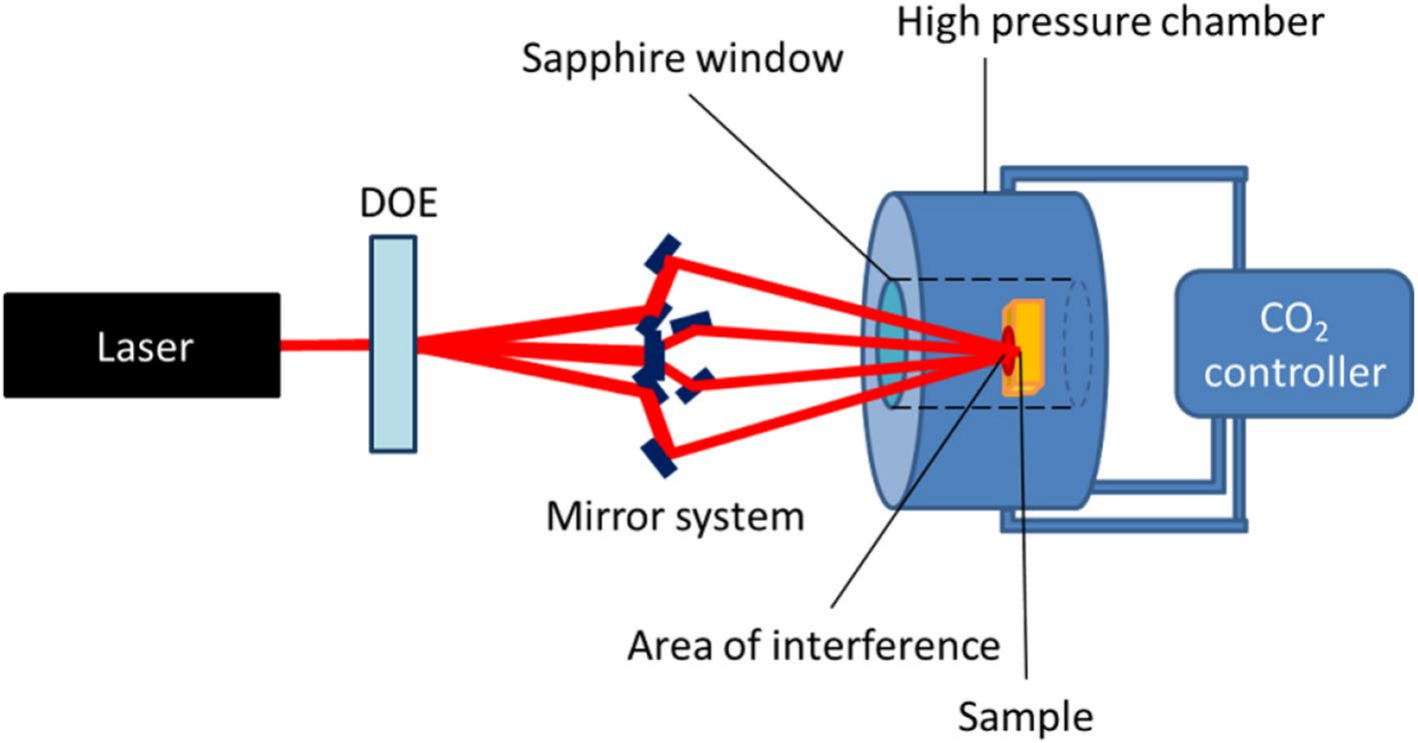
Schematic of experimental setup for direct laser interference patterning in high pressure CO_2_.

The experimental setup consisted of a laser, a high-pressure chamber, CO_2_ source and controller, and an optical system. The setup was arranged as described in the schematic in Fig. 4. The detailed description for the experimental setup is mentioned in our previous study. The laser and optical system design and its theory for interference, the description of the beam splitting, beam guiding for mirror-based, as well as of the lens-based interference system is described in detail in our previous publication^15^. For this study, only mirror-based DLIP was used to pattern the titanium and zinc samples.

A brief description of the setup is given below:

- **Laser**: A ns laser from Innolas Laser GmbH with pulse duration 10 ns, repetition rate 10 Hz, and energy 300 mJ/pulse was used to produce interference pattering on target surfaces. The laser beam output was top-hat energy distribution, offering possibility for almost uniform laser fluence. This laser offered long coherence length and low wave-front distortion. The beam diameter was 5 mm. A pulse picker was connected to the laser output, to set the number of pulses for each experiment to be one. Pulse picker used is a rotating disc that is synched to the laser pulse, so the single pulse is allowed. Other pulses are absorbed by the pulse picker.
- **High pressure chamber:** The autoclave was custom design from SciMed Limited (UK), made of SS316 steel, internal volume 28 mL, and suitable for pressure up to 300 bar and temperature up to 150 °C. It included two sapphire optical viewports of 35 mm diameter and 10 mm thickness, out of which only one is used for laser beam input into the chamber. The sapphire viewports were coated with anti-reflective coating (ion beam sputtered tantalum pentoxide and silicon dioxide), with measured transmission > 99.5% of original light. The chamber is fitted with thermocouple and pressure gauge to measure the temperature and pressure inside the chamber respectively.
- **CO**_**2**_** source and controller**: Carbon dioxide bottle containing ≥ 99.8% pure CO_2_ from AGA Oy Ab, acted as the CO_2_ source. The controller system consisting of a CO_2_ pump, a chiller, and a heater, was connected to a PLC controller to meticulously pump CO_2_ into the chamber at the desired rate, temperature, and pressure. Also connected is an automatic backpressure regulator (ABPR) to depressurize the chamber in a controlled manner.
- **Optical system**: The optical system consisted of a mirror-based laser interference system where the laser beam is divided to four beams using a DOE, followed by a mirror system that guides the four beams to cause interference at the target surface. The incidence angle and azimuth angle is defined by the position of the optical components. The azimuth angle of the system is 0;180,90 and 270 degrees. Incident angle during the test was 11 degrees.

### High pressure patterning of samples by mirror DLIP

For patterning, the titanium and zinc samples were cut from their respective thin metal sheets with femtosecond laser in order to avoid bending due to the cutting with a mechanical tool. All but the thicker Zn samples were cut with the laser. 120 µm thick Ti was used as it was delivered, as the surface quality was good enough for the processing. Thicker 700 µm thick Zn samples were polished before processing to a mirror finish, as in the previous tests with the 120 µm thick Zn sheet metal, it was noticed to have irregularities in the surface topography that may affect with the pattern. Thinner metal sheets (Ti, Zn with purity 99.99%) were acquired from Goodfellow Cambridge Ltd.

**Figure 5:**
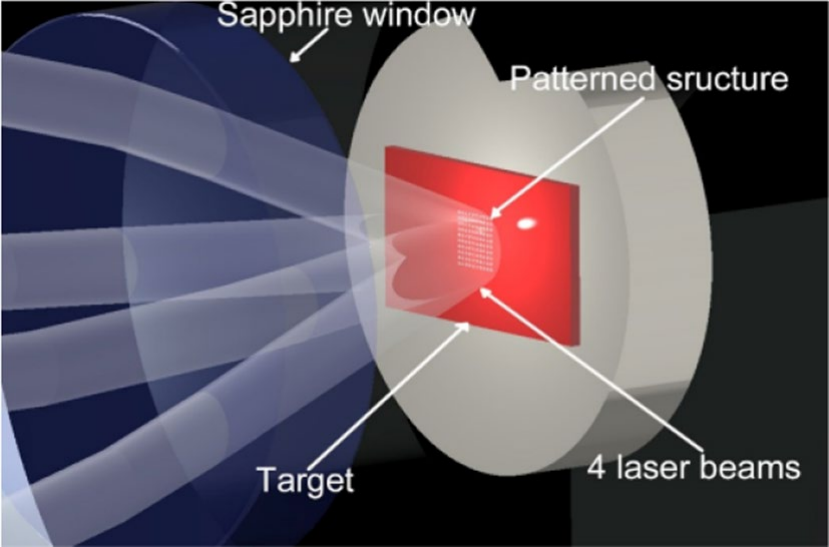
Schematic model for four-beam laser interference patterning in CO_2_ at 100 bar pressure.

Figure 5 shows the schematic model of patterning in supercritical and liquid CO_2_ at 100 bar pressure. For this, the samples were cut to about 23 × 10 mm pieces and mounted on the sample holder, so that the surface was as flat as possible for good results. The sample was clamped against the surface from the sides, leaving center area to be processed. After attaching the sample, the holder was placed inside chamber, and the chamber was closed by inserting and tightening the sapphire window into place. Then the optical setup is checked again to focus the beams on the target surface. This is implemented with two micrometer actuated position stages, that moves the chamber relative to the optical assembly. Before processing, the CO_2_ chamber was flushed with CO_2_ gas to reduce effect of ambient gases such as O_2_ and N_2_ and then CO_2_ was pumped and heated to a desired pressure and temperature. After the processing parameters were set, the pressure (100 bar) and temperature (35 °C or 27.5 °C, for supercritical or liquid state, respectively) of CO_2_ inside chamber is allowed to stabilize. When the desired CO_2_ temperature and pressure are reached, single laser pulse hits the target surface to cause interference patterning on an area of diameter around 4.5 mm (depending on the laser power). Pulse picker ensures single pulse patterning. After the patterning step is finished, the chamber is slowly de-pressurized to room conditions using ABPR and finally the sample is removed from the chamber.

#### Characterization methods

The patterned samples were studied using a field emission scanning electron microscope (FESEM Zeiss Ultraplus) at an acceleration voltage of 15 kV and for elemental analysis, the patterned surface was investigated using Energy dispersive X-ray spectroscopy (EDS Inca Energy 350). The donut ring thicknesses were measured from FESEM micrographs using ImageJ software (version 1.50i). The surface composition of the laser patterned samples were measured using X-ray photoelectron spectroscopy (XPS) and X-Ray induced auger electron spectroscopy (XAES). The lens-defined selected-area XPS was performed on ∅2 mm analysis area employing non-monochromatized DAR400 Al Kα X-ray source and Argus hemispherical electron spectrometer (Omicron Nanotechnology GmbH). The background subtracted XPS spectra were least-squares fitted with a minimum number of synthetic components to achieve good overall fitting of the spectra. The relative atomic concentrations were calculated using Scofield photoionization cross sections and an experimentally measured transmission function of the Argus analyser. For topography, the atomic force microscopy was performed using Bruker Dimension Icon in PeakForce tapping mode. The AFM data was analyzed using Gwyddion software version 2.61.

## Data Availability

The data used to support the findings of this study are included within the article.
